# Post-Diagnostic Beta Blocker Use and Prognosis of Ovarian Cancer: A Systematic Review and Meta-Analysis of 11 Cohort Studies With 20,274 Patients

**DOI:** 10.3389/fonc.2021.665617

**Published:** 2021-06-17

**Authors:** Zhao-Yan Wen, Song Gao, Ting-Ting Gong, Yu-Ting Jiang, Jia-Yu Zhang, Yu-Hong Zhao, Qi-Jun Wu

**Affiliations:** ^1^ Department of Clinical Epidemiology, Shengjing Hospital of China Medical University, Shenyang, China; ^2^ Clinical Research Center, Shengjing Hospital of China Medical University, Shenyang, China; ^3^ Department of Obstetrics and Gynecology, Shengjing Hospital of China Medical University, Shenyang, China

**Keywords:** beta blocker, meta-analysis, ovarian cancer, post-diagnostic, survival, systematic review

## Abstract

**Objectives:**

Previous experimental studies have indicated that exposure to beta blocker provides protective effects against ovarian cancer (OC). However, findings from epidemiologic studies have still been controversial. Therefore, we carried out a meta-analysis to update and quantify the correlation between post-diagnostic beta blocker usage and OC prognosis.

**Methods:**

The meta-analysis had been registered at PROSPEPO. The number of registration is CRD42020188806. A comprehensive search of available literatures in English prior to April 16, 2020, was conducted in PubMed, EMBASE, and the Web of Science databases. Random-effects models were used to calculate overall hazard ratios (HRs) and 95% confidence intervals (CIs). Publication bias assessments, and subgroup, sensitivity, and meta-regression analyses were also performed.

**Results:**

Of the 637 initially identified articles, 11 retrospective cohort studies with 20,274 OC patients were included. The summary HRs did not reveal any statistically significant associations between post-diagnostic beta blocker use and OC prognosis characteristics, such as total mortality (HR = 1.08, 95% CI = 0.92–1.27, *I^2^* = 76.5%, n = 9), cancer-specific mortality (HR = 1.22, 95% CI = 0.89–1.67, *I^2^* = 88.1%, n=3), and progression-free survival (HR = 0.88, 95% CI = 0.75–1.05, *I^2^* = 0, n = 4). No evidence of publication bias was observed in current analysis. In our subgroup analyses, the majority of results were consistent with the main findings. However, several positive correlations were detected in studies with ≥800 cases (HR = 1.20, 95% CI = 1.05–1.37), no immortal time bias (HR = 1.28, 95% CI = 1.10–1.49), and adjustment for comorbidity (HR = 1.20, 95% CI = 1.05–1.37). In the meta-regression analysis, no evidence of heterogeneity was detected in the subgroups according to study characteristics and confounding factors.

**Conclusions:**

Post-diagnostic beta blocker use has no statistical correlation with OC prognosis. More prospective cohort studies are necessary to further verify our results.

**Systematic Review Registration:**

Identifier (CRD42020188806).

## Introduction

Ovarian cancer (OC) is one of the deadliest cancer types of gynecological reproductive system, with over 200,000 new cases and 150,000 deaths globally in 2018 (1). Over 70% of OCs are discovered at advanced stages (stages III–IV) and with fast-growing metastases in the peritoneal cavity, largely because of unnoticed early symptoms and the lack of effective diagnostic approaches (2). For stage III and IV OC patients, survival rates of 5 years are only 42% and 26%, respectively (3). Therefore, some strategies that will have a beneficial impact on the treatment outcome of patients with OC should be developed to improve OC prognosis.

Beta blockers are medicines commonly used in a range of cardiovascular diseases, for example, coronary artery diseases or hypertension because of their function on the adrenergic system *via* inhibiting beta receptors (4, 5). Beta blocker treatment may prevent metastasis by inhibiting tumor cell invasion, tumor-associated inflammation, and vasculature remodeling to limit tumor cell dissemination (6–8). In recent years, vitro researches have indicated a positive correlation of beta blocker on the prognosis of OC (9–11). However, the differences in metabolism between human beings and other species preclude the extrapolation of data from cell and animal studies to human biology (12, 13).

In view of systematic review and meta-analysis, including available literatures up until September 2017, Yap et al. (14) was unable to show statistically relationship between beta blocker use (pre- and post-diagnostic) on OC prognosis (hazard ratio [HR] = 0.73, 95% confidence interval [CI] = 0.43–1.23). However, these findings were generated on the basis of a combined study of pre- and post-diagnosis beta blocker use on OC prognosis, which is not ideal because such an association could differ between these two phases. Majidi et al. (15) investigated the relationship between common medications and the survival of OC patients (including beta blockers) in a systematic review and meta-analysis of available literatures up until May 2019. The authors were unable to find a statistically significant relationship between beta blocker use and OC patients survival (HR = 0.97, 95% CI = 0.84–1.11) after adjusting for immortal time bias. However, this meta-analysis also explored mixed effects of pre- or post-diagnostic beta blocker use, and no further subgroup analyses, such as age at diagnosis, sample size, or study quality, were performed to explore possible sources of heterogeneity. Interestingly, results from a meta-analysis, including five studies (16–20) with 3,140 OC patients until September 2017 by Na et al. (21) revealed that use of post-diagnostic beta blocker was significantly related with improved survival of OC patients (HR = 0.59, 95% CI = 0.36–0.96). However, this meta-analysis had substantial heterogeneity (*I^2^* = 88.0%), which was not further explored through subgroup analyses.

During the last 2 years, many of high-quality studies with additional potential confounder adjustments have been published (22–25). Several studies have indicated that post-diagnostic beta blocker use increased OC mortality (22, 25), while other studies did not show an association (23, 24). In 2020, a large prognostic cohort study with 6,197 OC patients showed that post-diagnostic beta blocker use increased mortality (25). In contrast, no association was observed in a study by Harding et al. involving 2,195 OC patients (24). These inconsistencies could be attributed to the differences in the type of beta blockers used, the health status of OC patients, sample sizes, and adjustments for potential confounding factors. A more latest meta-analysis of existing evidence may further reveal the true effect of beta blocker in the treatment of OC. The source of heterogeneity has been further analyzed by subgroup, sensitivity, and meta-regression analyses. As far as we know, there are currently no published literatures comprehensively analyzing the data on post-diagnostic beta blocker use and OC survival. Consequently, we carried out an updated systematic review and meta-analysis of existing cohort studies to further identify the correlation between post-diagnostic beta blocker usage and OC prognosis.

## Methods

### Data Sources and Searches

The systematic review and meta-analysis was carried out in accordance with Preferred Reporting Items for Systemic Reviews and Meta-Analyses guidelines (26) and Meta-Analysis of Observational Studies in Epidemiology guidelines (27). Prior to study selection, the protocol of this meta-analysis was registered through the International Prospective Register of Systematic Reviews (registration number: CRD42020188806).

Two authors (Z-YW and T-TG) independently searched PubMed, EMBASE, and Web of Science databases for all relevant literatures published up until April 30, 2021. The following key words were included in our literature search: (“beta blocker” OR “beta blockers” OR “atenolol” OR “propranolol” OR “metoprolol” OR “arotinolol” OR “betaxolol” OR “bevantolol” OR “bisoprolol” OR “carteolol” OR “carvedilol” OR “celiprolol”) AND (“ovarian” OR “ovary”) AND (“neoplasm” OR “carcinoma” OR “cancer” OR “tumor”) AND (“survival” OR “mortality” OR “death”). The list of references included in the study was further screened to determine other publications.

### Study Selection

We put the retrieved citations into the reference management database and delete the literatures by automatic and manual methods. The judgment standard is based on the inclusion criteria determined before the search.

Studies that meet the following criteria will be included in our analysis (1): observational study or randomized controlled trial (2); exposure defined as use of beta blocker after diagnosis for OC patients (3); survival (such as disease-specific survival, progression-free survival, cancer-specific survival, overall survival) or mortality as outcome (such as all-cause mortality); and (4) analyses with HR or relative risk and 95% CI, or provided data suitable for risk estimates and 95% CI calculation.

Literatures, which meet the following criteria, were excluded (1): meeting abstracts, studies performed in animals, or non-original studies, including commentaries, editorials review articles, systemic reviews, meta-analyses (2); lack of sufficient risk estimates or related data in calculating risk estimates; and (3) studies were written in non-English.

### Data Extraction and Quality Assessment

Two authors (Z-YW and T-TG) extracted basic data of literatures by standardized form. Inconsistency was solved by a third author (J-YZ). The following data were extracted from the included articles: last name of first author, year of publication, country of origin, follow-up length, OC cases and events’ number, stage of cancer, classification of outcomes, and covariates matched in the study design or confounding factors adjusted in the primary statistical analysis.

In order to better identify the quality of included studies, we applied the Newcastle-Ottawa Scale (NOS) to evaluate quality of literatures for observational studies (28). The NOS scale comprises eight categories divided into three fields for selection, comparability, and outcome. In addition to the item Control for important factors or additional factors, each study could be given every item a maximum of one star. These studies, which obtained full stars in at least two classifications of selection, comparability, or outcome assessment, were classified as low risk of having bias (29, 30).

### Statistical Analysis

Since the differences in the populations and conditions among different studies might not exactly explain a common effect, a random-effects model was applied for summarizing HR in our analysis (30). The heterogeneity of all studies by calculating *I^2^* statistics, which assesses meta-analysis variability caused by differences between all studies instead of sampling errors (31). Using >75%, 50% to 75%, and <50% as the demarcation points indicated high, medium, and low levels of heterogeneity (31). We carried out subgroup analyses to probe into heterogeneous sources by using prespecified variables, for example, region, median number of cases, study quality, International Federation of Obstetrics and Gynecology (FIGO) stage of OC, length of follow-up, and adjustment for potential confounder factors such as age at diagnosis, stage of FIGO, comorbidities, residual diseases, histology, chemotherapy, and use of non-beta blocker drugs. We carried out sensitivity analyses to explore the impact of a single research on the summary HR *via* ruling out one research every time in the analysis (32). In addition, a meta-regression model was applied for identifying heterogeneous sources between subgroups. Finally, we evaluated publication bias through Egger’s linear regression (33), Begg’s rank correlation method (34), and visual inspection of funnel plots. All analyses were conducted using Stata version 11.0 software (StataCorp, College Station, TX). A two-tailed P value < 0.05 was regarded statistically significant.

## Results

### Search Results, Study Characteristics, and Quality Assessment

**Figure 1:**
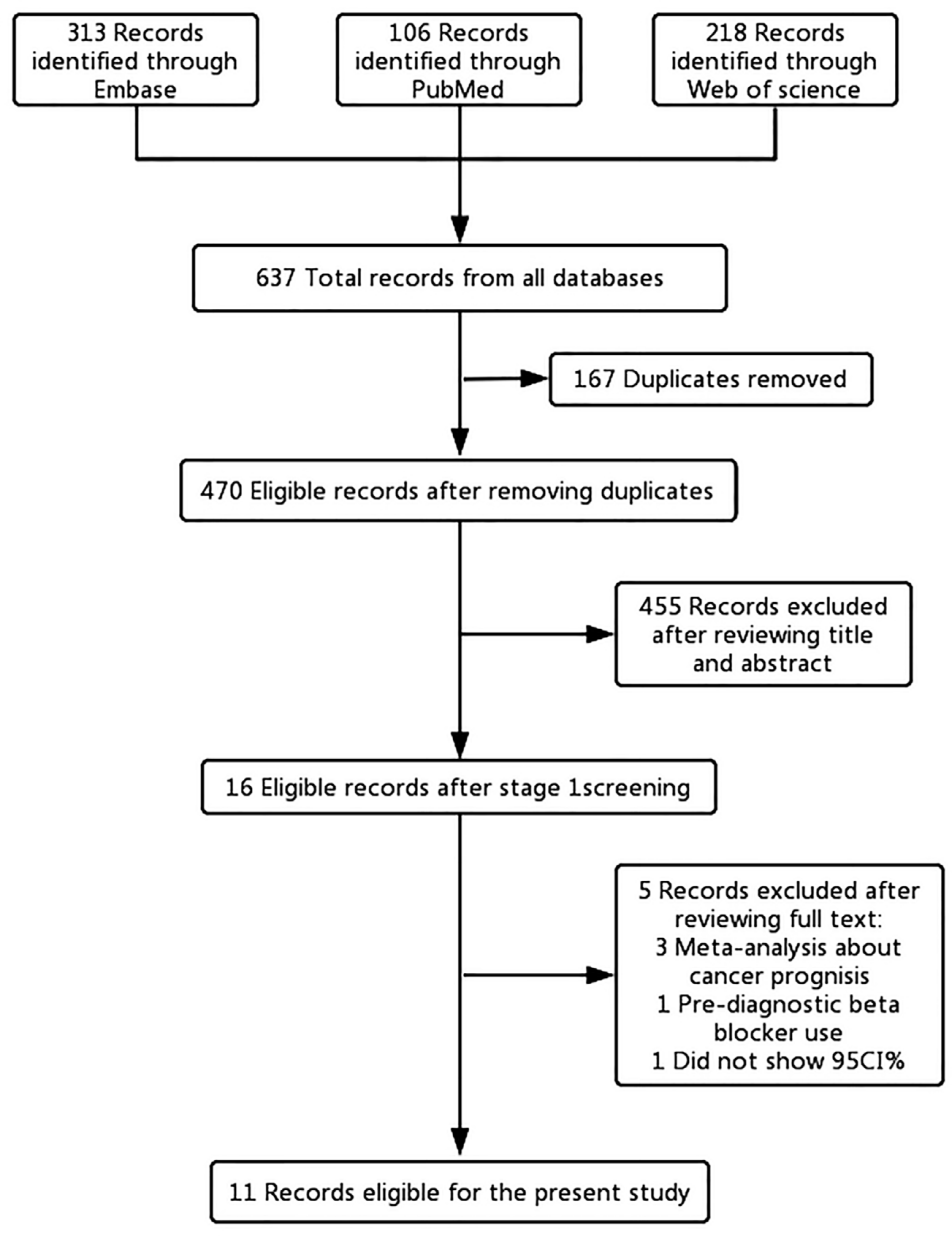
Flowchart of the study selection. The flowchart shows the process used to select studies for our meta-analyses focusing on the association between post-diagnostic beta blocker use and ovarian cancer (OC) prognosis.

Of the 637 articles that were reviewed, 621 were removed following review of the titles and abstracts. After full-text screening of the remaining 16 articles, five articles (14, 15, 21, 35, 36) were excluded. Two of the excluded articles (14, 21) examined the impact of beta-blockers on cancer prognosis, and one article (15) analyzed the relationship between common medications and survival of OC patients. The remaining two articles were excluded because one article (35) did not provide the 95% CI and one article (36) included pre-diagnostic beta blocker use for OC prognosis. In the end, 11 published literatures were included for the main analysis (**Figure 1**).

**Table 1** reveals the major features of the included literatures. All of the included literatures were published between 2012 and 2020, with a scope of 123 to 6,626 OC cases each study. Five of the studies were performed in the United States (17, 18, 20, 22, 24), three of the studies were performed in Europe (16, 25, 38), two studies were performed in Korea (23, 37), and one study was conducted (19) across Belgium, Canada, and Germany. All of the cohort studies were retrospectively designed, with a median follow-up duration between 17 and 91 months. **Table 2** outlines the potential confounder adjustments in the preliminary analysis of the included literatures. Majorities of the studies adjusted for potentially important confounder factors, such as age at diagnosis (n = 10) and FIGO stage (n = 8). A limited number of studies adjusted for comorbidity (n=6), histology (n=4), chemotherapy use (n = 3), residual disease (n = 3), race (n = 3), and grade (n = 3).

**Table 1:** Characteristics of included studies.

First author, reference, year	Country	No. of cases/event	Patient stage	Follow-up	Outcome
Cho et al. (23), 2020	Korea	878/470	I–IV	33.9 months (median)	Progression-free survival
Gonzalez et al. (22), 2020	USA	534/NA	III–IV	32 months (median)	Overall survival
Couttenier et al. (25), 2019	Belgium	6197/2918	I–IV	3.49 years (median)	All-cause morality Cancer-specific mortality
Harding et al. (24), 2019	USA	2195/796	I–IV	2.2 years (median)	Cancer-specific mortality
Baek et al. (37), 2018	Korea	866/381	NA	6.35 years (median)	Overall survival
Al-Niaimi et al. (17), 2016	USA	185/123	I–IV	91 months(median)	Progression-free survival Overall survival
Heitz et al. (16), 2016	Germany	801/682	I–IV	40 months(median)	Progression-free survival Overall survival
Watkins et al. (18), 2015	USA	1425/NA	III–IV	44.9 months (median)	Overall survival Disease-specific survival
Johannesdottir et al. (38), 2013	Denmark	6626/NA	NA	2.55 years (median)	All-cause mortality
Heitz et al. (19), 2013	Germany Belgium Canada	381/267	NA	17 months (median)	Progression-free survival Overall survival
Diaz et al. (20), 2012	USA	248/NA	III–IV	27 months(median)	Overall survival

NA, not available.

**Table 2:** Adjustment potential confounders of included studies.

First author, reference, year	Adjustment for potential confounders in the primary analysis
Cho et al. (23), 2020	Age, FIGO stage, histologic type, residual disease status after PDS, platinum resistance, comorbidity, cytoreduction status
Gonzalez et al. (22), 2020	Age, race, CCI, FIGO stage, PDS vs NACT, residual disease status, statin use, metformin use, aspirin use
Couttenier et al. (25), 2019	Age at diagnosis, year of diagnosis, FIGO stage, grade, cancer histology, diabetes, and pulmonary comorbidities
Harding et al. (24), 2019	Age at diagnosis, year of diagnosis, race/ethnicity, marital status, census tract poverty level, location of residence, tumor histology, FIGO stage at diagnosis, receipt of surgery, receipt of chemotherapy, Charlson comorbidity score, diagnosis of diabetes and hypertension
Baek et al. (37), 2018	N/A
Al-Niaimi et al. (17), 2016	Age, stage, grade, cytoreduction status, BMI, presence or absence of diabetes
Heitz et al. (16), 2016	Age, ECOG, Charlson comorbidity score, tumor residuals, histology, BMI, FIGO stage
Watkins et al. (18), 2015	Age, race, stage, BMI, neoadjuvant therapy, diabetes, hypertension
Johannesdottir et al. (38), 2013	Age, comorbidity level, prior use of diuretics, year of diagnosis, aspirin, statins
Heitz et al. (19), 2013	Age, platinum free-interval, study treatment arms, ECOG performance status
Diaz et al. (20), 2012	Age, FIGO stage, grade, suboptimal cytoreduction

BMI, body mass index; CCI, Charlson comorbidity index; ECOG, Eastern Cooperative Oncology Group; FIGO, International Federation of Gynecology and Obstetrics; N/A, not available; NACT, neoadjuvant chemotherapy; PDS, primary debulking surgery.

The results the quality assessment in view of the NOS are summarized in **Table 3**. One study (19) was graded as high risk, and 10 studies (16–18, 20, 22–25, 37, 38) were graded as low risk. For “selection” classification, three studies (16, 17, 20) were not assigned to full stars. For the item of “control for important factor or additional factor”, two studies (19, 37) were not awarded two stars since these studies had adjusted for less than two important confounder factors. For classification of “outcome”, one study (19) was not assigned to full stars because of insufficient duration of follow-up.

**Table 3:** Methodological quality of cohort studies included in the meta-analysis.

First author, reference, publication year	Selection				Comparability	Outcome			Risk of bias^d^
	Representativeness of the exposed cohort	Selection of the unexposed cohort	Ascertainment of exposure	Outcome of interest not present at start of study	Control for important factor or additional factor^a^	Assessment of outcome	Follow-up long enough for outcomes to occur^b^	Adequacy of follow-up of cohorts^c^	
Cho et al. (23), 2020	*	*	*	*	**	*	*	*	Low risk
Gonzalez et al. (22), 2020	*	*	*	*	**	*	*	*	Low risk
Couttenier et al. (25), 2019	*	*	*	*	**	*	*	*	Low risk
Harding et al. (24), 2019	*	*	*	*	**	*	*	*	Low risk
Baek et al. (37), 2018	*	*	*	*	–	*	*	*	Low risk
Al-Niaimi et al. (17), 2016	*	–	*	*	**	*	*	*	Low risk
Heitz et al. (16), 2016	–	*	–	*	**	*	*	*	Low risk
Watkins et al. (18), 2015	*	*	*	*	**	*	*	*	Low risk
Johannesdottir et al. (38), 2013	*	*	*	*	**	*	*	*	Low risk
Heitz et al. (19), 2013	*	*	*	*	*	*	–	*	High risk
Diaz et al. (20), 2012	–	*	*	*	**	*	*	*	Low risk

*A study could be awarded a maximum of one star for each item except for the item Control for important factor or additional factor.

The definition/explanation of the Newcastle-Ottawa Scale in each column is available from http://www.ohri.ca/programs/clinical_epidemiology/oxford.asp.

a A maximum of two stars could be awarded for this item. Studies that controlled for age at diagnosis received one star, whereas studies that controlled for other important confounders such as FIGO stage, comorbidity received an additional star.

b A cohort study with a follow-up time >24 months was assigned one star.

c A cohort study with a follow-up rate >75% was assigned one star.

d Studies that obtained a full scores at least two domains were considered to have a low risk of bias, other situations were considered as high risk.

### Association of Post-Diagnostic Beta Blocker Usage With OC Prognosis

**Figure 2:**
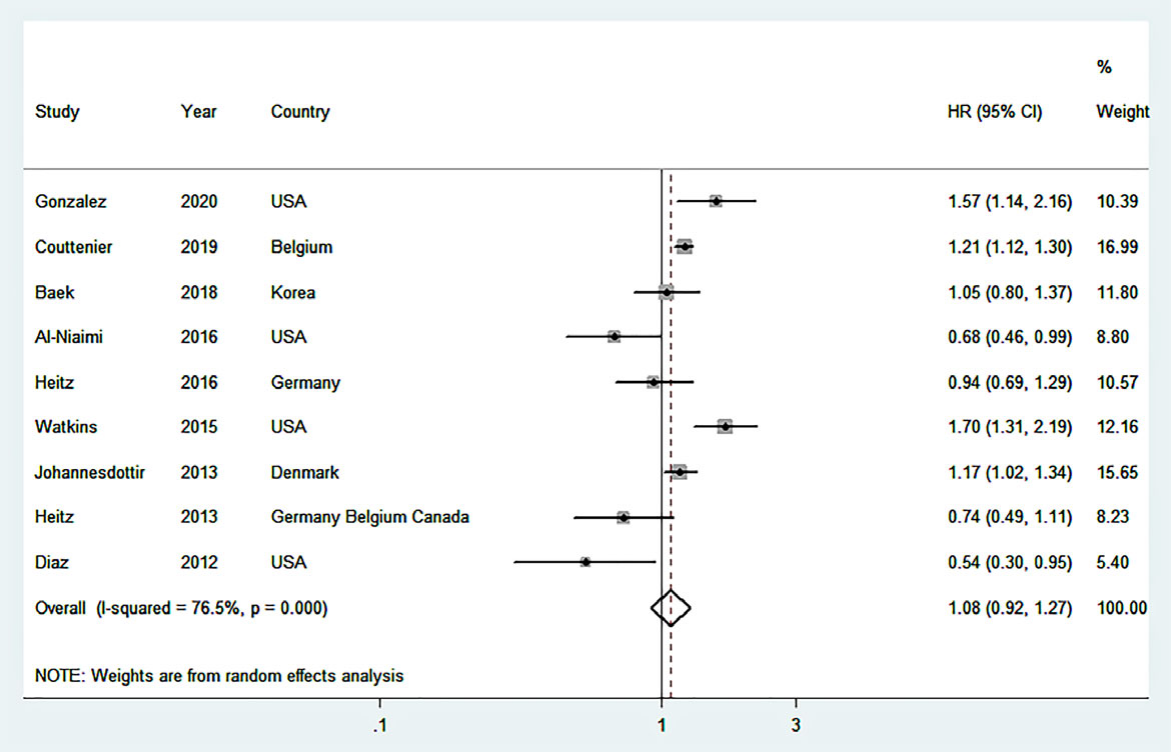
Forest plot (random-effects model) of the association between post-diagnostic beta blocker use and total mortality of OC patients. Squares indicate study-specific hazard ratios (HR), where the size of the square reflects the study-specific statistical weight; horizontal lines indicate the 95% confidence interval (CI); diamonds denote the summary hazard ratio with 95% CI.

Nine studies (16–20, 22, 25, 37, 38), including 17,201 OC patients and 4,433 events were used to estimate the summary relationship between post-diagnostic beta blocker usage and total mortality of OC patients. The overall HR was 1.08 (95% CI = 0.92–1.27) with high heterogeneity (*I* ^2^ = 76.5%) (**Figure 2**). No publication bias was discovered (**Supplementary Figure S1**) (Egger’s P = 0.22 and Begg’s P = 0.05). Three (18, 24, 25) studies were applied for assessing the overall effects of post-diagnostic beta blocker usage on cancer-specific mortality (HR=1.22, 95%CI=0.89-1.67, *I* ^2^ = 88.1%), and four (16, 17, 19, 23) studies were applied for assessing the overall effects of post-diagnostic beta blocker use on progression-free survival (HR = 0.88, 95% CI = 0.75–1.05, *I* ^2^ = 0%).

The results of subgroup and meta-regression analyses are presented in **Table 4**. Most of these findings were consistent with the main results. Notably, in the stratified analysis, we observed significant positive associations in studies with ≥800 cases (HR = 1.20, 95% CI = 1.05–1.37), no immortal time bias (HR = 1.28, 95% CI = 1.10–1.49) and adjustment for comorbidity (HR = 1.20, 95% CI = 1.05–1.37). Furthermore, no evidence of heterogeneity source was detected in meta-regression model on the base of the variables mentioned.

**Table 4:** Summary risk estimates of the association between post-diagnostic beta blocker use and prognosis of ovarian cancer (user vs. non-user).

	No. of study	HR (95%CI)	*I^2^(%)*	P*	P**
**Total mortality**	9	1.08 (0.92–1.27)	76.5	<0.01	
**Cancer-specific mortality**	3	1.22 (0.89–1.67)	88.1	<0.01	
**Progression-free survival**	4	0.88 (0.75–1.05)	0	0.773	
**Subgroup analyses for total mortality**					
**Type of beta blocker**					0.877
Non-selective beta blocker	3	1.24 (0.90–1.71)	68.9	0.04	
Selective beta blocker	4	1.21 (0.94–1.55)	76.0	0.006	
**Region**					0.888
USA	4	1.03 (0.61–1.74)	88.1	<0.01	
Non-USA	5	1.10 (0.98–1.24)	50.6	0.088	
**Number of cases**					0.183
<800	4	0.83 (0.51–1.36)	82.9	0.001	
≥800	5	1.20 (1.05–1.37)	61.9	0.033	
**Study quality**					0.375
Low risk	8	1.12 (0.95–1.31)	75.8	<0.01	
High risk	1	0.74 (0.49–1.11)	N/A	N/A	
**FIGO stage**					0.535
I-IV (All)	3	0.96 (0.68–1.34)	80.7	0.006	
III-IV (Advanced)	3	1.21 (0.71–2.05)	84.6	0.002	
**Immortal time bias**					0.118
Yes	6	0.90 (0.68–1.19)	72.3	0.003	
No	3	1.28 (1.10–1.49)	70.5	0.034	
**Adjustment for potential confounders**					
**Age at diagnosis**					0.984
Yes	8	1.08 (0.90–1.29)	79.0	<0.01	
No	1	1.05 (0.80–1.37)	NA	NA	
**FIGO stage**					0.778
Yes	6	1.09 (0.84–1.40)	82.1	<0.01	
No	3	1.03 (0.83–1.29)	55.6	0.105	
**Comorbidity**					0.289
Yes	4	1.20 (1.07–1.34)	42.7	0.155	
No	5	0.90 (0.60–1.35)	84.8	<0.01	
**Residual disease**					0.541
Yes	2	1.21 (0.73–2.01)	80.2	0.025	
No	7	1.04 (0.87–1.25)	79.3	<0.01	
**Histology**					0.880
Yes	2	1.12 (0.89–1.41)	57.8	0.124	
No	7	1.04 (0.81–1.34)	80.9	<0.01	
**Chemotherapy**					0.262
Yes	1	1.57 (1.14–2.16)	N/A	N/A	
No	8	1.03 (0.87–1.22)	77.3	<0.01	
**Non-beta blocker drug use**					0.276
Yes	2	1.31 (0.99–1.73)	63.7	0.097	
No	7	0.98 (0.78–1,23)	80.5	<0.01	

*P-Value for heterogeneity within each subgroup. **P-Value for heterogeneity between subgroups with meta-regression analysis.

When each study was removed from the major analysis in turn, the summary results did not change substantially in the sensitivity analysis (**Supplementary Figure S2**). The resulting HR of total mortality ranged from a low of 1.03 (95% CI = 0.82–1.29; *I* ^2 =^ 78.7%) after removing the study by Couttenier et al. (25) to a high of 1.14 (95%CI = 0.97–1.33; *I* ^2 =^ 73.0%) after removing the study by Al-Niaimi et al. (17).

## Discussion

The present updated meta-analysis based on eleven cohort studies, including 20,274 OC patients and 5,699 events were unable to observe a significant association between post-diagnostic beta blocker use and OC prognosis. These null findings were consistently detected in numerous subgroup and sensitivity analyses. To our knowledge, the current analysis is the most comprehensive meta-analysis of the available cohort studies estimating the aforementioned relationship between post-diagnostic beta blocker use and OC prognosis.

Compared with two previous published systematic reviews and meta-analyses (14, 15), our present study revealed consistent results. Yap et al. (14) indicated no statistical association between beta blocker use on OC prognosis. Majidi et al. (15) was also unable to find a statistically significant relationship between beta blocker use and OC patients’ survival. These two reports had common limitations in exploring mixed effects of pre- or post-diagnostic beta blocker use and failed to provide information regarding subgroup analyses stratified by geographic locations, study quality and whether adjustments were made for confounders. The present meta-analysis included several relevant high-quality studies (22–25) with additional potential confounder adjustments during the past 2 years. To explore sources of study heterogeneity, subgroup analysis and meta-regression analysis were conducted based on study characteristics and confounding factors. Further investigation of relationships between beta blocker use and total mortality, cancer-specific mortality and progression-free survival as outcomes revealed no significant correlation.

Although we were unable to observe significant result in the subgroup analysis stratified by geographic location, the point estimate was slightly different (1.03 for U.S. vs. 1.10 for non-U.S.). This observation could partly be attributed to different rates of beta blocker use in OC patients. For example, Watkins et al. (18) reported that the usage rate was 18.9% in the U.S. based on 1,425 OC patients from 2004 to 2010, whereas Couttenier et al. (25) reported this rate as 38.3% in Belgium based on 6,197 OC patients from 2004 to 2014.

In the subgroup analysis layered by immortal time bias, we detected positive correlation between use of beta blocker after diagnosis and total mortality for OC patients in studies without immortal time bias. The probability of accidental results could not be excluded because limited studies were included in this subgroup analysis. Immortal time bias was first described by Suissa (39), and refers to drug usage classification based on data about usage of beta blocker after the beginning of follow-up. Herein, OC patients had to survive until follow-up before they would to be defined as exposed group. This immortal time bias can result in overestimating survival rate of the exposed group and may interpret why survival rate of beta blocker users has increased. For example, two studies (17, 20) reported improved survival among beta blocker users. However, both results were probably affected by immortal time bias. On the contrary, three other studies (18, 25, 38) avoided immortal time bias in the study design and showed no evidence that the use of beta blocker was beneficial for ovarian survival. Therefore, future primary studies should address this issue of immortal time bias.

Beta blocker are generally prescribed for hypertension. OC patients with hypertension were more likely to take beta blocker as prescription drugs, whereas OC patients without hypertension may also use beta blocker for perioperative treatment. Baek et al. (37) described a increasement in survival with post-diagnostic beta blocker use among OC patients with hypertension (HR = 0.65, 95% CI = 0.45–0.93); however, no statistically correlated was found for OC patients and normal blood pressure levels (HR = 0.60, 95% CI = 0.34–1.07). Therefore, there may be differences in underlying disease, which could be a source of bias for the resulting analysis. In addition, there could also be confounding variables associated with the severity of the health status during treatment, if OC patients with high blood pressure also has comparatively more serious malignant tumors, they are less likely to keep on using beta blocker following diagnosis (40). Information on stage and grade are not widely obtained and do not grasp the whole condition of the severity of disease. Therefore, a low evaluation of the mortality for beta blocker users could have happened.

Clinical studies presented that the use of beta blocker has been confirmed to decrease total mortality and tumor proliferation in some malignant tumors (41, 42). The underlying biological mechanisms by which beta blocker use after diagnosis may alter the survival rate of those with OC have been explored in certain researches. Beta blockers exert antineoplastic effects on the cyclic adenosine monophosphate pathway, causing down regulation of adhesion receptors and upregulation of tumor suppressor genes (43). In addition, beta blockers decrease expression of the pro-proliferative protein Ki-67 and pro-survival protein Bcl-2 and increase expression of pro-apoptotic p53 protein expression (44). Studies have shown that OC patients with better social support produce less vascular endothelial growth factor (VEGF) (11). Orepinephrine, epinephrine, and isoproterenol significantly increased VEGF production *via* SKOV3 cells, and the effects of these pathways were inhibited by the beta blocker called propranolol (11). Selective beta blockers inhibited phosphorylation of the mitogenic signaling regulators p44/42 MAPK, p38 MAPK, JNK, and CREB, and promoted phosphorylation of the cell survival/apoptosis regulators AKT, p53, and GSK3β42 (45).

The advantages of this study deserve to be emphasized. This study investigated the association between different exposures (type of post-diagnostic beta blocker use) and outcomes (total mortality, cancer-specific mortality, progression-free survival) of OC patients. In consideration of study features and main adjustments for confounding variables, subgroup, sensitivity, meta-regression analyses were conducted to probe into possible sources of heterogeneity. Moreover, majorities of the included articles had a low risk of bias after using the NOS to assess the literature quality of all included articles.

The disadvantages of this study deserve to be outlined. First, we relied on prescription records to define medication use without information on compliance. This method could not make an accurate evaluation of medications used if patients are prescribed medication but not taking these drugs. Second, included cohort studies were all retrospective, which may endure potential risks of information and selection biases. This could lead to exposure group selection if inappropriate and if information is incomplete. Third, because of the features of observational studies, it is not enough to rule out the possibility of residual confounding effects of incomplete or unmeasured factors. Post-diagnostic beta blocker use is usually related to different clinical and non-clinical factors, for example, age at diagnosis, cancer stage, comorbidities, chemotherapy administration, and use of non-beta blocker drugs. However, not all of the currently included studies have adjusted for the abovementioned potential confounder factors (37). Fourth, due to limited information supplied by the included literatures, we were unable to determine the categories (beta-1 blocker on cardiac ventricular myocytes, beta-2 blocker on bronchial epithelium and systemic vascular, and beta-3 blocker on adipocytes), dose, intensity, duration of post-diagnostic beta blocker use and OC prognosis in our meta-analysis. Finally, we merely included and analyzed openly published literatures; however, other unpublished literatures and gray literatures content with our criteria might have been ignored.

## Conclusions

The current meta-analysis reveals that post-diagnostic beta blocker use does not have an association with prognosis of OC patients. Further large-scale, prospective, and randomized controlled trials are necessary to verify the therapeutic effect of beta blockers and other factors, such as intensity, dose, duration, and type of beta blocker use on OC patients.

## Data Availability Statement

The data analyzed in this study are subject to the following licenses/restrictions: The data that support the findings of this study are available from the corresponding author upon reasonable request. Requests to access these datasets should be directed to wuqj@sj-hospital.org.

## Author Contributions

Q-JW and Y-HZ conceived and designed the study. Z-YW and T-TG performed the literature search and reviewed the search results for study inclusion. Z-YW and Y-TJ performed the data curation and formal analysis. Z-YW, SG, T-TG, Y-TJ, and J-YZ participated in writing the original draft. Z-YW and SG contributed equally to this work. All authors contributed to the article and approved the submitted version.

## Funding

This study was supported by grants from the National Key R&D Program of China (2017YFC0907401 to Y-HZ), the Natural Science Foundation of China (82073647 and 81602918 to Q-JW), the China Postdoctoral Science Foundation Funded Project (2018M641752 to Q-JW), the LiaoNing Revitalization Talents Program (XLYC1907102 to Q-JW and XLYC1802095 to Y-HZ), Shenyang high level innovative talents support program (RC190484 to Q-JW), and 345 Talent Project to Q-JW (M0268).

## Conflict of Interest

The authors declare that the research was conducted in the absence of any commercial or financial relationships that could be construed as a potential conflict of interest.
