# Supplementary material for: Post-Diagnostic Beta Blocker Use and Prognosis of Ovarian Cancer: A Systematic Review and Meta-Analysis of 11 Cohort Studies With 20,274 Patients
Source: Front Oncol. 2021 Jun 17;11:665617. doi: 10.3389/fonc.2021.665617 (PMC8247638; doi:10.3389/fonc.2021.665617)
Supplement: Supplementary file 1 [file DataSheet_1.docx]

**Supplementary materials**

**
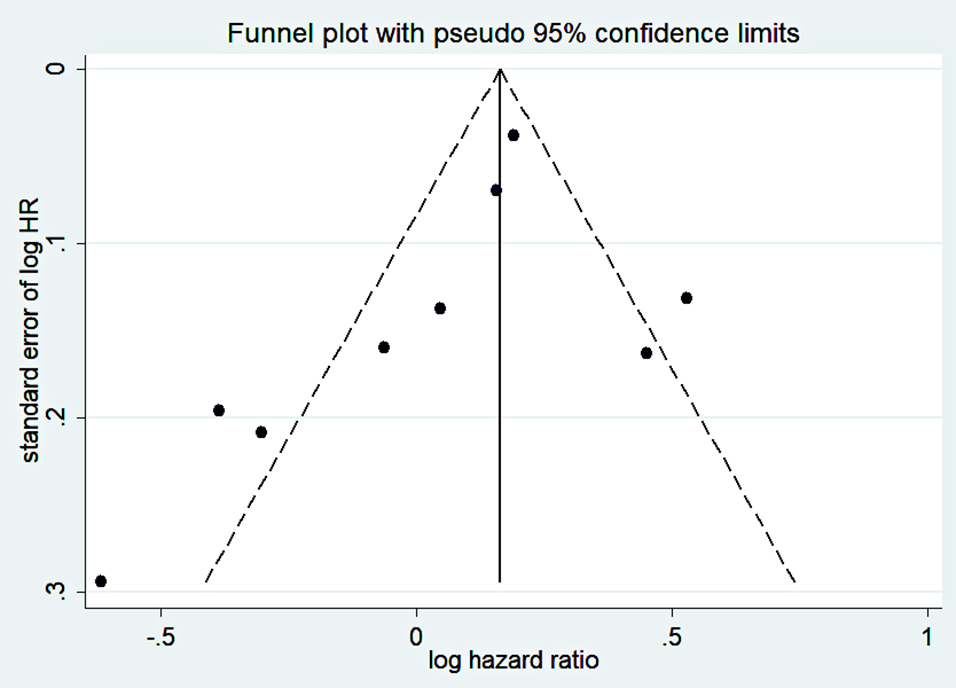
**

**Sumplemental Figure S1**. Funnel plot of publication biases of studies included in our meta-analysis focusing on the association between post-diagnostic beta blocker use and total mortality of OC patients. Each dot represents an individual study.

**
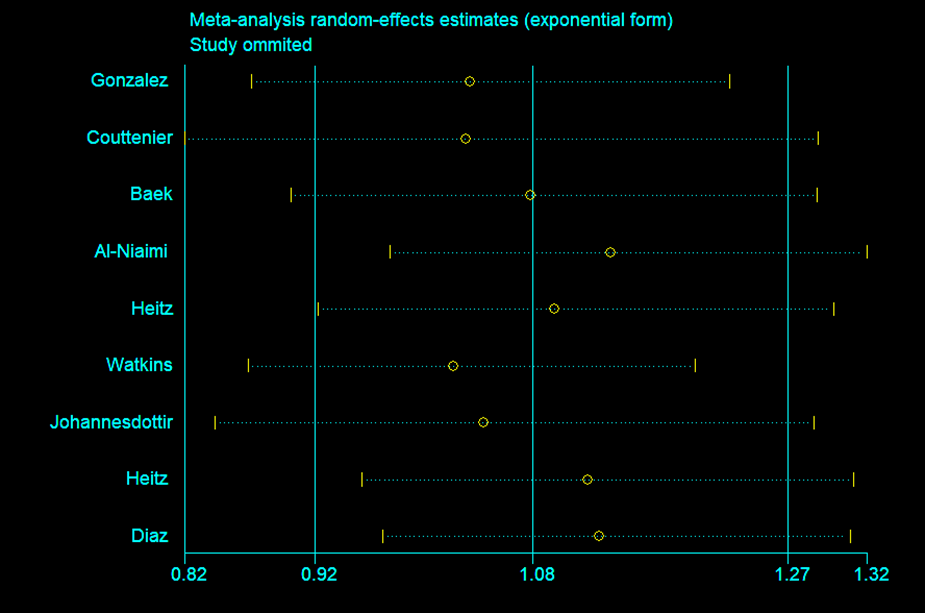
**

**Sumplemental Figure S2**. A sensitivity analysis was performed by removing each study in turn and recalculating the pooled odds hazard ratio.
